# Bio-inspired vision mimetics toward next-generation collision-avoidance automation

**DOI:** 10.1016/j.xinn.2022.100368

**Published:** 2022-12-29

**Authors:** Gary J.W. Xu, Kun Guo, Seop Hyeong Park, Poly Z.H. Sun, Aiguo Song

**Affiliations:** 1 Human Factors in Driving Lab, Huawei Technologies Co., Ltd., Shenzhen 518038, China; 2 School of Psychology, University of Lincoln, Lincoln LN6 7TS, UK; 3 School of Software, Hallym University, Chunchon Gangwon-do 24252, South Korea; 4 School of Mechanical Engineering, Shanghai Jiao Tong University, Shanghai 200240, China; 5 School of Instrument Science and Engineering, Southeast University, Nanjing 210096, China

## Motivation

The current “deep learning + large-scale data + strong supervised labeling” technology framework of collision avoidance for ground robots and aerial drones is becoming saturated. Its development gradually faces challenges from real open-scene applications, including small data, weak annotation, and cross-scene. Inspired by the neural structure and processes underlying human cognition (eg, human visual, auditory, and tactile systems) and the knowledge learned from daily driving tasks, a high-level cognitive system is developed for integrating collision sensing and collision avoidance. This bio-inspired cognitive approach has the advantages of good robustness, high self-adaptability, and low computation consumption in practical driving scenes.

## LGMD for collision detection

Vision is the primary sense for motion perception. It provides key information for navigation, course control, gaze stabilization, and other motion-related activities.^1^ Biological visual neural networks have evolved over millions of years and are working efficiently in nature. These visual neural networks can be ideal models for designing artificial vision systems, especially for collision avoidance. As a visual neural structure, the lobula giant movement detector (LGMD) neuron in locusts shows a strong ability to detect moving objects.^2^ This LGMD neuron consists of a four-layer neural network in which the first layer detects the changes in luminance, the second and third layers process the interaction between excitation and inhibition responses, and the last layer outputs a spike (peak response). When the locust eyes perceive an approaching object, the LGMD neuron will be stimulated to output a spike signal to warn the locust of potential danger at distance. The high sensitivity of LGMD neurons in perceiving objects in motion^3^ is suitable to promote vehicles to detect the approaching object.

**Figure 1:**
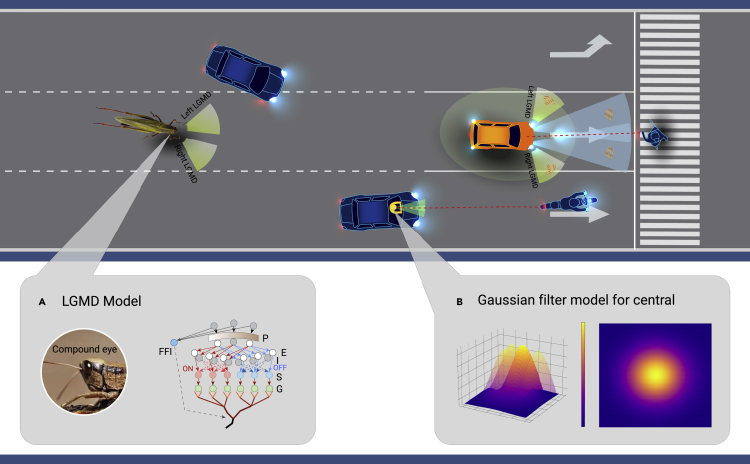
Binocular LGMD-based obstacle-avoidance strategy considering human factors

To take advantage of bio-inspired vision in collision detection, in this commentary, an innovative visual neural network that possesses similar collision selectivity of LGMD neurons is reported. The modeling of LGMD is shown in Figure 1A. The visual stimuli are perceived from the photoreceptor (P) layer to the excitation (E) and inhibition (I) layers, then the visual signals are decomposed into ON/OFF channels and fused separately in the summation (S) layer, and the grouping (G) layer is further aggregated for the output. The visual signals input to ON channels indicate the increased brightness in its navigation, and vice versa. When an obstacle approaches, visual stimulation will activate the LGMD neuron to detect potential collisions. The locust will then update its behavior to avoid collisions.

## Human factors in driving

This commentary discusses the impact of two human factors on driving: central fixation bias and binocular vision. So far, only a few studies have been conducted thoroughly on modeling human factors in driving and investigating its potential in ground robotics and aerial drones.

### Central fixation bias

Central fixation bias, which is a key factor in driving tasks as evidenced in Xu et al.,^4^ particularly for experienced drivers, is not thoroughly reflected in previous research. It is reported that human vision exerts a tendency of central fixation bias to actively search the gist of approaching scenes and bind visual features for higher cognitive understanding; that is, the visual information from the central visual field/region is paid more attention while that from outer regions is paid less attention.^3^ Once potential hazards approach, the drivers’ gaze or attention distribution exhibits high selectivity at the central visual area to enhance their alertness. To reflect this selectivity of gaze distribution in the obstacle detection process, our reported LGMD considers the effect of central fixation bias in receiving visual stimuli and transmitting the processed signals to the P layer. Specifically, the Gaussian filter shown in Figure 1B is incorporated into LGMD to keep the central region of the image as clear as in the original image while blurring the outer regions of the image. This in turn sharpens the sensitivity of LGMD to forward motion.

### Binocular vision

Another human factor in driving, binocular vision, should also be fully considered. Compared with monocular vision, binocular vision could fuse depth measurement in navigation, which is conducive to accurate perception of obstacle distance. Different from most of the existing LGMD models^1^^,^^5^ that only verified the collision detection performance under monocular vision, our commentary provides the collision detection performance of LGMDs under binocular vision.

## Collision avoidance strategy

Based on the above discussion on LGMD and the two human factors in driving, a collision avoidance strategy is reported, which is inspired by the visual-based obstacle avoidance mechanism of locusts and considers two human factors of central fixation bias and binocular vision. In this strategy, central fixation bias is modeled and placed in front of the P layer of LGMD. Two LGMDs, left and right LGMDs, are adapted to process the images of the left and right cameras in front of the vehicle, respectively.

During the motion of the object, once the collision is detected by either the left or right LGMD, two procedures of direction selection and collision body distance prediction are triggered simultaneously. In the direction selection procedure, the values of normalized membrane potentials (NMPs) output by the left and right LGMD models are compared. The side with a larger NMP value is then selected. In the collision body distance prediction program, NMP and feedforward inhibition of the left and right LGMD models and the current robot motion speed are fed into the trained multi-layer perceptron model. Then, the model will output the predicted distance. Based on the direction selection and distance prediction, the best motor control is finally derived for optimizing the steering selection of collision avoidance under the emergent situation.

## Summary and future perspective

LGMD is a promising bio-inspired model for collision avoidance. In addition to its superior performance, this kind of bio-inspired vision mimetics approach can reduce the dependence of algorithms on graphics processing units and central processing units, which becomes its unique dominance for next-generation automation. The next step of the bio-inspired vision metrics approach should focus on the processing of road scenes with complex backgrounds. On real roads, different weather and lighting conditions will lead to large variance in visual appearance of driving scenes, which is a difficult challenge that should be further studied.

Besides, human factors, particularly in the attention process, are crucial for safe driving. Like human driving, after a quick scan through the entire visual field, LGMD tends to select a target area (ie, focus of attention) and then invests more attention resources to extract detailed information about the target and suppress irrelevant surrounding information, which is the primary foundation of situation awareness. This allows us to use limited attention resources to quickly screen out high-value targets from a large amount of information. As a survival mechanism evolved in human evolution, the visual attention mechanism considerably improves the efficiency of visual information processing. This key block would further enhance the performance of ground robots and aerial drones guided by human factors in driving.
